# Factors associated with participation in life situations in people with COPD

**DOI:** 10.1177/14799731221079305

**Published:** 2022-03-05

**Authors:** Cassandra D’Amore, Sachi O’Hoski, Lauren E Griffith, Julie Richardson, Roger S Goldstein, Marla K Beauchamp

**Affiliations:** 1 School of Rehabilitation Science, 62703 McMaster University, Hamilton, ON, Canada; 2 27375 West Park Healthcare Centre, Toronto, ON, Canada; 3 Department of Health Research Methods, Evidence, and Impact, 62703 McMaster University, Hamilton, ON, Canada; 4 Department of Medicine, University of Toronto, Toronto, ON, Canada; 5 Department of Physical Therapy, University of Toronto, Toronto, ON, Canada

**Keywords:** Chronic lung disease, community participation, disability, pulmonary rehabilitation, social role

## Abstract

**Objective:**

To examine potential determinants of participation frequency and limitations in people with Chronic Obstructive Pulmonary Disease (COPD).

**Methods:**

For this secondary analysis, we grouped the following factors using the International Classification of Functioning, Disability and Health (ICF) components: age, psychological distress (Hospital Anxiety and Depression Scale (HADS)), gait aid use, supplemental oxygen use, grip strength, modified Medical Research Council Dyspnea scale, Short Physical Performance Battery, and Six-Minute Walk Test (6MWT). Participation was measured using the frequency and limitation domains of the Late Life Disability Instrument (LLDI). Relationships between factors and participation were examined using linear regression.

**Results:**

Ninety-six participants (age 68.7 ± 8.1 yrs; FEV_1_ %pred 34 IQR 25–54) were included in the analysis. Factors were linked to four ICF components: activity, body functions, personal, and environmental factors. The final model for LLDI-frequency contained HADS, use of gait aid, and 6MWT (F (3, 81) = 27.69 (*p* < .001), R^2^ = 0.51), and for LLDI-limitations, the final model included age, HADS, and 6MWT (F (3, 82) = 19.74 (*p* < .001), R^2^ = 0.42).

**Discussion:**

Participation in life situations in people with COPD is associated with multiple ICF components. Psychological distress (i.e., anxiety and depression symptoms) and mobility were important determinants of participation frequency and limitations. Prospective studies are needed to confirm these relationships.

## Introduction

The World Health Organization (WHO) defines participation as the involvement in a life situation and recognizes that experiencing restrictions in participation leads to disability.^1^ Participation, as defined by the WHO, includes physical, mental, and social activities, covering several different domains such as communication, domestic life, self care, and interpersonal interactions and relationships.^2^ According to the WHO’s International Classification of Functioning, Disability and Health (ICF), participation can be influenced by the interaction among several factors from the other ICF components (i.e., groups of factors), including activities, body functions and structures, and environmental and personal factors.^1^ In older adults, participation has been linked to both functional ability and survival^3^ and can influence overall quality of life,^4^ making it an important consideration for healthcare professionals and policy makers.^5^

Since the release of the ICF, literature confirming the importance of participation has increased. However, limited research remains on the specific factors from different ICF components that influence participation levels, particularly in clinical populations. People diagnosed with Chronic Obstructive Pulmonary Disease (COPD) experience considerable limitations in mobility^6^ and distressing disease-specific symptoms such as shortness of breath and exercise intolerance^7^ that make them a high-risk group for experiencing participation restrictions. Although pulmonary rehabilitation programs are considered the standard of care for symptom management in patients with COPD, current programs focus primarily on ICF body functions (e.g., muscle strength) and activities (e.g., walking) but do not specifically target participation. Identification of factors that influence participation could inform how to modify existing pulmonary rehabilitation programs to actively target not just physical function limitations but also disability in life situations.

This study aimed to examine the relationship between ICF factors commonly measured during pulmonary rehabilitation and participation in life situations among people with COPD. This study had three objectives (1) to examine relationships between individual factors and participation, (2) to examine the relationships between different ICF components and participation, and (3) to identify the combination of factors (from all ICF components) that explained the most variation in participation.

## Methods

### Study design and participants

This was a secondary analysis of a cross-sectional study by O’Hoski et al.^8^ (2020) that recruited consecutive eligible participants from two hospitals with respirology programs in Ontario, Canada. To be included in the study participants had to be diagnosed with COPD, have at least a 10 pack-year smoking history, and be non-institutionalized. Participants were excluded if they had comorbidities that caused severe disability distinct from their COPD or experienced a language barrier that prevented them from completing the assessments. Participants provided written informed consent prior to assessment. All participants from the primary study were included in this analysis. We followed the STROBE reporting guidelines for cross-sectional studies (checklist available in Supplemental Appendix 1).^9^

### Primary measures

We used the Late Life Disability Instrument (LLDI) to measure participation in life situations. The development of the LLDI was informed by Nagi’s disablement model and is also consistent with the ICF.^1,10,11^ The LLDI contains two domains; the frequency domain examines how often a person reports they participate in different situations (what people report they actually do) and the limitation domain assesses reported level of limitation in the ability to participate (what people report they can do).^10,12^ Both domains consist of 16 questions that are scored from one to five based on the level of agreement with the prompt “How often do you do a particular activity” or “To what extent do you feel limited in doing this particular activity” for the frequency and limitation domains, respectively.^12^ These questions cover a variety of life situations such as keeping in touch with others via phone or email, taking care of one’s health (e.g., managing medications and scheduling appointments), taking part in organized social activities, and taking part in a regular fitness program.^12^ Total scores are calculated and then scaled from 0 to 100, where higher scores represent greater participation frequency or fewer reported limitations.^10,12^ The LLDI is the only instrument that has been validated as a measure of participation in people with COPD.^8^ Both the frequency and limitation domains demonstrated strong test-retest reliability (ICC_2,1_ 0.9 SEM 2.49 points, ICC_2,1_ 0.9 SEM 4.20 points, respectively) and fair to moderate correlations with physical function and quality of life in people with COPD.^8^

### Factors

**Figure 1.**
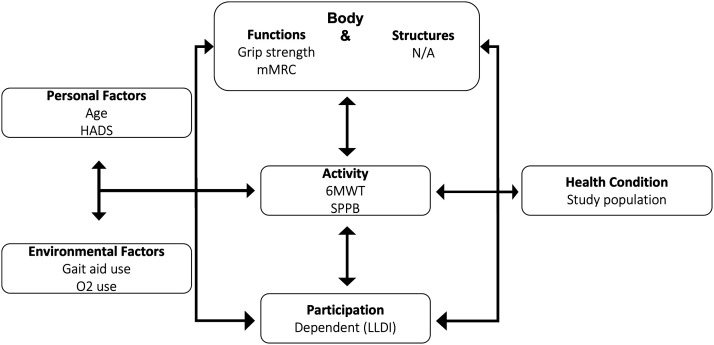
Mapping potentials factors in COPD on to the International Classification of Functioning, Disability and Health (ICF). HADS (Hospital Anxiety and Depression scale), LLDI (Late Life Disability Instrument), 6MWT (Six-Minute Walk Test), SPPB (Short Physical Performance Battery), mMRC (modified Medical Research Council Dyspnea scale).

We selected eight potential factors (i.e., independent variables) related to participation based on clinical experience and previous work in similar populations. Following the updated linking rules presented by Cieza et al. (2005), potential factors were grouped into the most relevant ICF component (activity, body functions and structures, personal factors, and environmental factors), and where appropriate, linked to a specific chapter (i.e., a subgroup within a component).^13^ For example, the Short Physical Performance Battery (SPPB) was included to capture mobility, specifically the ability to change positions or transfer. The authors, therefore, grouped SPPB under the activity component, specifically chapter d4. Mobility. ICF components and chapters were reviewed using the online ICF browser.^14^ Details from the linking process can be found in Supplemental Appendix 2. The eight potential factors related to participation were distributed across four ICF components; activity, body functions and structures, environmental factors, and personal factors (see Figure 1).

#### Activity

Two performance-based measures were linked to the activity component of the ICF as both represented meaningful concepts of mobility: the Six-Minute Walk Test (6MWT) and the SPPB. The 6MWT has been used to evaluate functional exercise capacity or more specifically the maximum distance a person can walk in 6-min^15^ The 6MWT was administered according to the American Thoracic Society Guidelines.^16^ Participants completed two tests, and the greater distance (meters, m) was recorded; greater distance covered indicates greater functional exercise capacity.^15^ The 6MWT distance has demonstrated strong construct validity and test-retest reliability in adults with chronic respiratory disease.^15^ The SPPB consists of three tasks: standing balance, repeated sit to stand, and a four-m gait speed test.^17^ Each task is given a score from 0 to 4 and summed for a score out of 12; higher scores represent better physical performance.^17^ Among people with COPD, the SPPB total score can discriminate between people with and without mobility limitations.^18^

#### Body functions and structures

Grip strength and breathlessness using the modified Medical Research Council Dyspnea Scale (mMRC) were included in this component. Grip strength is commonly used as a proxy measure for overall muscle strength in older adults.^19^ We used the average score from two trials with the dominant hand, and elbow flexed to 90°. A greater number of pounds (lb) indicates greater grip strength or greater overall strength. Grip strength measured with a dynamometer has good relative test-retest reliability (ICC >0.8) in at-risk older adults.^20^ The mMRC instructs participants to select a grade from 0 (not troubled by breathlessness except on strenuous exercise) to 4 (too breathless to leave the house, or breathless when dressing or undressing)^7^ to evaluate the degree to which dyspnea (i.e., the symptom of breathlessness) impacts daily activities. In people experiencing breathlessness, the mMRC has demonstrated excellent inter-observer agreement (98%).^21^

#### Environmental factors

Gait aid use and need for supplemental oxygen (O_2_ use) were included as environmental factors. The use of a gait aid was operationalized according to where it was used (i.e., in the home, in the community, both, and not at all). Similarly, oxygen use was operationalized by when oxygen was required (i.e., at rest, during exertion, and not at all/only when sleeping).

#### Personal factors

We included two personal factors: age of the participant in years and a measure of psychological distress. The Hospital Anxiety and Depression Scale (HADS) was originally designed to screen for anxiety and depression in inpatients but is now regularly used in the general public.^22,23^ It consists of 14 questions (7 for anxiety and 7 for depression), and each item is rated 0–3 with total scores ranging from 0 to 42.^22^ This unidimensional model (i.e., using total score) has been recommended as a measure of overall psychological distress, where higher scores represent greater severity of symptoms.^24,25^

### Statistical analysis

All statistical analyses were completed using STATA IC15(StataCorp LLC, College Station, TX). Demographic variables were summarized using mean and standard deviation (SD) for continuous variables (median and inter-quartile range (IQR) were substituted where appropriate) and frequency and proportions were used for categorical variables. Separate analyses were completed with each domain of the LLDI as the dependent variable. First, univariable linear regression was conducted for each potential factor. Second, multivariable regression was completed for each ICF component (i.e., all factors that were grouped under that component were entered into the model). Third, a multivariable analysis including all potential factors, from all ICF components, was completed using backwards stepwise regression (factors were removed from the model at *p* > .05). Both the full models and final models are presented below. Due to sample size constraints, for the final analyses all categorical variables were dichotomized. Assumptions were confirmed (e.g., Breusch–Pagan test for heteroskedasticity, Shapiro–Wilk W test, and variance inflation) and potential outliers were investigated for each model. A missing data analysis was completed by examining patterns of missingness; where greater than 5% of values were missing a sensitivity analysis was completed. Individuals with missing values were compared to those with values, using an independent t-test, and where appropriate, a Wilcoxon rank sum test across various demographic variables. Ten imputed datasets were created using multivariate normal distribution (seed 1234) to create estimates for adjusted models.

## Results

### Demographic information

The sample consisted of 96 participants with a mean age of 68.7 years (SD = 8.1), 43 (44.8%) of whom were female. Over 50% of participants were in either GOLD category III or IV (severe or very severe) for disease severity; the median FEV_1_ percent predicted of the sample was 34 (IQR 25, 54). See Table 1 for participant characteristics. On average, participants had a lower score in the frequency domain than the limitation domain of the LLDI; 47.0 (SD = 5.1) and 58.6 (SD = 9.6), respectively.

**Table 1.** Participant characteristics (*n* = 96).

Characteristics	Mean (standard deviation)
Age (years)	68.73 (8.13)
Sex, female, *n* (%)	43 (44.8)
FEV_1_ %pred, median IQR	34 (25, 54)
GOLD stage, *n* (%)	
Mild (I)	6 (6.5)
Moderate (II)	19 (20.4)
Severe (III)	33 (35.5)
Very severe (IV)	35 (37.6)
Supplemental oxygen, uses, *n* (%)	51 (53.1)
Gait aid use, uses, *n* (%)	43 (45.0)
Dyspnea, mMRC, *n* (%)	
0	3 (3.5)
1	24 (25.0)
2	30 (32.3)
3	30 (32.3)
4	9 (9.4)
6MWT (meters)	305.90 (102.99)
Grip strength (pounds)	60.67 (23.78)
SPPB, median (IQR)	9 (7, 10)
HADS–total score	13.74 (7.21)
LLDI–limitation domain	58.62 (9.58)
LLDI–frequency domain	47.02 (5.13)

mMRC (modified Medical Research Council Dyspnea scale), 6MWT (Six-Minute Walk Test),SPPB (Short Physical Performance Battery), HADS (Hospital Anxiety and Depression scale), LLDI (Late Life Disability Instrument).

### Perceived frequency of participation

Five individual factors were significantly related to the LLDI frequency domain in the univariable analysis; mMRC (R^2^ = 0.15, *p* = .006), SPPB (R^2^ = 0.12, *p* < .001), HADS (R^2^ = 0.14, *p* < .001), O_2_ use (R^2^ = 0.14, *p* = .001), and 6MWT (R^2^ = 0.30, *p* < .001). We found, that for a one-m increase in 6MWT distance, participation frequency score increased by 0.03-points. Whereas, for psychological distress, every 1-point increase on the HADS was associated with a 0.27 decrease in the participation score. When we examined each ICF component on its own, all component models were significant (see Table 2).

**Table 2.** Regression models for frequency in participation.

Independent variable	a) Univariable analysis			b) ICF component models			c) Final adjusted model	
	β	95% CI		β	95% CI		β	95% CI
							R^2^ = 0.42	*p* < .001
Activity component				R^2^ = 0.30	*p* < .001			
6MWT	R^2^ = 0.30 0.03	*p* < .001 (0.02, 0.04)		0.02	(0.01, 0.04)		0.03	(0.02, 0.04)
SPPB	R^2^ = 0.12 0.92	*p* < .001 (0.41, 1.42)		0.25	(–0.32, 0.83)			
Body functions component				R^2^ = 0.15	*p* = .011			
Grip strength	R^2^ = 0.03 0.04	*p* = .11 (−0.008, 0.08)		0.02	(–0.03, 0.06)			
mMRC	R^2^ = 0.15	*p* = .006						
Grade 1	−5.05	(−10.93, 0.85)		−4.68	(–10.71, 1.36)			
Grade 2	−7.14	(–12.97, −1.31)		−6.60	(–12.68, −0.53)			
Grade 3	−7.23	(–13.05, −1.40)		−6.66	(–12.74, −0.59)			
Grade 4	−10.81	(–17.23, −4.40)		−10.27	(–16.91, −3.62)			
Personal factors				R^2^ = 0.17	*p* < .001			
HADS	R^2^ = 0.14 −0.27	*p* < .001 (−0.42, −0.13)		−0.31	(–0.46, −0.17)		−0.15	(–0.28, −0.01)
Age	R^2^ = < 0.001 −0.02	*p* = .820 (−0.14, 0.11)		−0.11	(–0.24, 0.02)			
Environmental factors				R^2^ = 0.14	*p* = .020			
Gait aid use	R^2^ = 0.02	*p* = .550						
In the community	−0.66	()		−0.85	(–2.38, 2.21)		2.41	(0.40, 4.42)
In the home	−4.36	0.244		−1.61	(–8.80, 5.57)			
Both	−1.55	0.366		−0.51	(–3.79, 2.77)			
O_2_ use	R^2^ = 0.14	*p* = .001						
At rest	−3.80	(−5.95, −1.65)		−3.65	(–5.94, −1.36)			
During exertion	0.38	(–2.46, 3.23)		0.39	(–2.55, 3.34)			

In analysis c categorical variables were operationalized as follows: Gait aid use = yes/no, O2 use = yes/no, mMRC = 0–2/3–4. 6MWT (Six-Minute Walk Test), SPPB (Short Physical Performance Battery), mMRC (modified Medical Research Council Dyspnea scale), HADS (Hospital Anxiety and Depression scale).

The model with all factors entered explained 45% of the variance in participation frequency (F (8, 77) = 7.86 (*p* < .001)). Using backwards stepwise regression, 6MWT (β = 0.03 (0.02, 0.04)), gait aid use (β = 2.41 (0.40, 4.42)), and HADS (β = –0.15 (−0.28, 0.01)) remained in the final model (Table 2). Assumptions were met for all models and no outliers were identified. Adjusting for use of a gait aid and HADS did not change the relationship between 6MWT and participation frequency (i.e., 1-m increase equals 0.03 increase in score). However, after adjusting for mobility (6MWT) and gait aid use, every 1-point increase in the psychological distress outcome (HADS) resulted in a 0.15 decrease in participation frequency.

### Perceived limitations of participation

All individual factors were significantly associated with the LLDI limitation scale, with R^2^ values ranging from 0.05 (*p* = .030) for age to 0.31 (*p* < .001) for HADS. We found that a 1-point increase in HADS resulted in a 0.72 decrease in participation limitations score (i.e., greater limitation). Examining models for each ICF component, we found that all components significantly explained a portion of the variance in limitation scores (see Table 3 for details).

**Table 3.** Regression models for limitation in participation.

Independent variable	a) Univariable analysis			b) ICF component models			c) Final adjusted model	
	β	*95% CI*		β	95% CI		β	95% CI
							R^2^ = 0.51	*p*<0.001
Activity component				R^2^ = 0.28	P < .001			
6MWT	R^2^ = 0.28 0.05	P < .001 (0.03, 0.06)		0.04	(0.02, 0.06)		0.04	(0.03, 0.06)
SPPB	R^2^ = 0.14 1.72	P < .001 (0.84, 2.60)		0.47	(–0.56, 1.51)			
Body functions component				R^2^ = 0.26	P < .001			
Grip strength	R^2^ = 0.10 0.12**	P = .002 (0.04, 0.19)		0.07	(–0.0002, 0.15)			
mMRC	R^2^ = 0.23	P < .001						
Grade 1	−11.61	(−21.53, −1.69)		−9.92	(–19.87, 0.04)			
Grade 2	−17.64	(–27.47, −7.82)		−15.11	(–25.15, −5.08)			
Grade 3	−17.99	(–27.80, −8.18)		−15.36	(–25.39, −5.33)			
Grade 4	−22.30	(–33.10, −11.50)		−19.78	(–30.75, −8.81)			
Personal factors				R^2^ = 0.31	P < .001			
HADS	R^2^ = 0.31−0.72	P < .001 (−0.95, −0.49)		−0.69	(–0.93, −0.45)		−0.45	(–0.69, −0.22)
Age	R^2^ = 0.05 0.24	P = .030 (0.02, 0.48)		−0.08	(–0.14, 0.29)		0.25	(0.06, 0.44)
Environmental factors				R^2^ = 0.21	P < .001			
Gait aid use	R^2^ = 0.10	P = .026 (−8.47, −0.47)		−2. 49	(–6.83, 0.94)			
In the community	−4.47*	(–25.83, −0.69)		−8.57	(–20.73, 3.60)			
In the home	−13.26*	(–10.99, 0.59)		−3.40	(–8.96, 2.16)			
Both	−5.20							
O_2_ use	R^2^ = 0.18	P < .001						
At rest	−8.30	(−12.04, −4.57)		−7.09	(–10.98, −3.20)			
During exertion	−4.30	(–9.24, 0.64)		−3.66	(–8.66, 1.34)			

In analysis c categorical variables were operationalized as follows: Gait aid use = yes/no, O2 use = yes/no, mMRC = 0–2/3–4. One datum point was determined to be an outlier by its influence over normality and removed from all analyses of limitation in participation. 6MWT (Six-Minute Walk Test), SPPB (Short Physical Performance Battery), mMRC (modified Medical Research Council Dyspnea scale), and HADS (Hospital Anxiety and Depression scale).

When we included all eight factors in the model, 54% of the variation in participation limitation scores was explained (F (8, 76) = 11.22 (*p* < .001)). After backwards stepwise regression only 6MWT (β = 0.04 (0.03, 0.06)), HADS (β = –0.45 (−0.69, 0.22)), and age (β = 0.25 (0.06, 0.44)) remained in the final model (Table 3). A 1-point increase on the psychological distress scale (HADS), adjusting for mobility (6MWT) and age, resulted in a decrease of 0.45 in the participation limitation score. According to our results, if age and HADS remain constant; a 1-m increase during the 6MWT would result in a 0.04-point increase in the participation limitation score. In the analysis with the participation limitation domain, one value was determined to be an outlier and removed from all analyses with this domain.

### Missing data analysis

After examining patterns of missingness, it was identified that only three outcomes had missing values (grip strength *n* = 1, 6MWT *n* = 3, and HADS *n* = 7). Due to 6% of participants missing HADS scores, a sensitive analysis was completed. Between group comparisons were completed for age, FEV_1_ percent predicted, sex, 6MWT, and both LLDI domains for those who did and did not complete the HADS for psychological distress. There were no significant differences found between those who completed HADS and those who did not. Multiple imputation were performed and estimates for adjusted models were carried out; however, this did not materially alter our models substantially.

## Discussion

To our knowledge, this is the first study to examine factors affecting life participation in people with COPD using a validated measure. The complexity of participation is highlighted in our results by the number of factors associated and the breadth of ICF components represented. Given the importance of participation as a health outcome, understanding the factors that influence participation is critical. Our findings highlight that both distance walked in 6-min and psychological distress are key determinants of participation frequency and limitation in people with COPD. We also found that different factors were associated with each participation domain (i.e., what people perceive they actually do vs. what they perceive they can do). Given the known importance of participation, and its association with the 6MWT and mental health as demonstrated in this study among people with COPD, participation should be considered as part of a comprehensive outcomes assessment in pulmonary rehabilitation.

### Summary of main findings

Our finding that multiple ICF components are related to both the frequency and limitation domains of the LLDI extends the existing literature on participation in older adults and in those with COPD.^3,26,27^ Our findings generally agreed with those of a similar study by Arnadottir et al. in a sample of 186 older adults (mean age 74 years old), of whom 65% were diagnosed with at least three medical conditions; they found LLDI scores of 47.7 (SD = 5.5) and 78.7 (SD = 15.8) for frequency and limitation, respectively. However, their final adjusted models included a greater number of ICF components compared to ours. This may be explained by the difference in population and a greater variety and number of factors that could be used because of their larger sample size. However, although our models contained fewer factors, they were able to explain a similar to greater portion of the variance in both participation domains in our sample of patients with COPD.

Our finding that psychological distress and mobility measured as distance walked in 6-min were important contributors to the adjusted model for both participation domains is consistent with previous literature and the clinical picture of this population. People with COPD have an increased risk for mobility limitations^6^ and anxiety and depression.^28^ These relationships align with clinical knowledge: increases in distance walked are associated with increased participation frequency and fewer limitations, and, increases in symptoms of psychological distress are associated with decreased participation frequency and increased limitations. Recently, a small cross-sectional analysis of 47 patients with COPD (FEV_1_ %pred 49.5 (SD = 18.1), 10% using O_2_) also found the 6MWT (median 460.9, IQR 384–512.0) to be a determinant of participation; however, the authors used a measure of participation that has not been previously validated in COPD in their study.^26^ Consistent with our findings of the importance of psychological distress, literature in the general older adult population shows that health status and depression symptoms can influence participation.^3,29^ Improving mobility is already a central tenant in pulmonary rehabilitation, the potential of these programs to promote participation is promising.^7^ On the other hand, in most pulmonary rehabilitation programs there is limited focus on mental health; this is likely an important target if existing programs are modified with the goal of improving participation. As noted in the Global Strategy for Diagnosis, Management, and Prevention of Chronic Obstructive Lung Disease, anxiety and depression are associated with worse clinical outcomes.^7^ Therefore, a more concerted effort to improve the symptoms of psychological distress is critical both for improving a patient’s clinical picture and based on our findings, participation in life roles. Clinicians and policy makers should consider increasing the priority placed on the management of these symptoms in treatment plans and resource funding.

Our results reflect the importance of evaluating what a person reports they are actually doing (i.e., frequency) in addition to what a person believes they can do (i.e., limitations) when assessing participation.^30^ Previous literature has shown that the frequency and limitation domains of the LLDI are two separate constructs of participation.^30^ Our study further supports this by showing different relationships between factors associated with each participation domain. Although the study of participation in older adults by Arnadottir et al. also found different factors associated with each LLDI domain, in contrast to our results, their data explained a greater relative portion of the variance in the frequency versus limitation domain.^29^ This difference may be explained by the use of a dichotomous frequency domain score in Arnadottir et al. compared to our use of the continuous scaled score.^29^ Nevertheless, these data suggest that interventions may need to be tailored to the individual participation domain of interest. Importantly, it is the self-reported frequency of participation that has the strongest predictive validity for adverse health outcomes in older adults, and therefore is an important domain to target in future work.^30^

### Limitations

This paper presents novel work on determinants of participation in COPD using a conceptually sound and well validated outcome measure, the LLDI. However, we would like to address a few limitations of this analysis. As a secondary analysis, the factors available for inclusion and sample size were predetermined. In our analysis, sample size did not allow us to include all the factors of interest available, and there may also have been important factors not available for selection. We circumvented this by referring to relationships found in previous literature involving similar populations (e.g., general older adults) to inform our factor selection. We restricted our independent variables (i.e., factors), using the one-ten rule of thumb suggested by Harris (1985).^31^ However, we did not account for dummy variables which resulted in us altering the operationalization of categorical variables before the final step in the analysis. We also acknowledge a common pitfall in exploratory analyses, the decrease in power and alpha due to the multiple tests. As a result, we need to be cautious when interpreting these results, and results should not be generalized outside of this population. Finally, as there was no healthy control group in this analysis, we cannot determine how the diagnosis of COPD impacts participation, but only which factors influence participation in this population. Despite these limitations, our study still provides valuable information on relationships that can inform future research on participation.

## Conclusion

This study demonstrates the importance of considering more than just physical impairments when trying to understand participation in life situations among people with COPD. Prospective studies are warranted to confirm the impact of psychological health and functional exercise capacity on participation in people with COPD.

## Supplemental Material

sj-pdf-1-crd-10.1177_14799731221079305 – Supplemental material for Factors associated with participation in life situations in people with COPD Click here for additional data file. Supplemental material, sj-pdf-1-crd-10.1177_14799731221079305 for Factors associated with participation in life situations in people with COPD by Cassandra D’Amore, Sachi O’Hoski, Lauren E Griffith, Julie Richardson, Roger S Goldstein and Marla K Beauchamp in Chronic Respiratory Disease
